# A Review of Potentially Toxic Elements in Sediment, Water, and Aquatic Species from the River Ecosystems

**DOI:** 10.3390/toxics13010026

**Published:** 2024-12-31

**Authors:** Md Muzammel Hossain, Iffat Jahan, Mudasir A. Dar, Maruti J. Dhanavade, Al Fattah Bin Mamtaz, Stephen J. Maxwell, Song Han, Daochen Zhu

**Affiliations:** 1 Biofuels Institute, School of Environment and Safety Engineering, Jiangsu University, Zhenjiang 212013, China; 1000006431@ujs.edu.cn (M.M.H.); muddar7@gmail.com (M.A.D.); hansong@ujs.edu.cn (S.H.); 2 Jiangsu Collaborative Innovation Center of Technology and Material of Water Treatment, Suzhou University of Science and Technology, Suzhou 215009, China; 3 Biodiversity Conservation and Fisheries Research Center, Dhaka 1207, Bangladesh; ch18032@mbstu.ac.bd; 4 Department of Chemistry, Mawlana Bhashani Science and Technology University, Santosh 1902, Bangladesh; 5 Department of Microbiology, Bharati Vidyapeeth’s Dr Patangrao Kadam Mahavidyalaya, Sangli 416416, India; marutijd@gmail.com; 6 Institute of Agribusiness & Development Studies, Bangladesh Agricultural University, Mymensingh 2202, Bangladesh; 7 College of Science and Engineering, James Cook University, Cairns, QLD 4878, Australia; stephen.maxwell@my.jcu.edu.au

**Keywords:** PTE, river, risk assessment, anthropological activities, monitoring

## Abstract

There is concern over potential toxic elements (PTEs) impacting river ecosystems due to human and industrial activities. The river’s water, sediment, and aquatic life are all severely affected by the release of chemical and urban waste. PTE concentrations in sediment, water, and aquatic species from river ecosystems are reported in this review. Among the PTEs, chromium (Cr), cadmium (Cd), lead (Pb), and nickel (Ni) revealed high pollution levels in water and aquatic species (fish and shellfish) at many rivers. The Karnaphuli, Ganga, and Lee rivers have high levels of Pb and Cd contamination, while the Buriganga and Korotoa rivers’ water had notable Ni contamination. A number of rivers with PTEs showed ecological risk as a consequence of the sediment’s potential ecological risk (PER), the pollutant load index (PLI), and the geoaccumulation index (Igeo). A comprehensive study suggests elevated PLI values in river sediments, indicating significant pollution levels, particularly in the Buriganga River sediment, marked by high Igeo values. The PER of the Shitalakshya and Buriganga rivers was marked as very high risk, with an E^i^_r_ > 320, while the Dhaleshwari and Khiru rivers showed ‘high risk’, with 160 = E^i^_r_ < 320. It was found that fish and shellfish from the Buriganga, Turag, and Swat rivers have a high concentration of Cr. PTE pollution across several river sites could pose health toxicity risks to humans through the consumption of aquatic species. The CR value shows the carcinogenic risk to human health from eating fish and shellfish, whereas an HI value > 1 suggests no carcinogenic risk. The occurrence of other PTEs, including manganese (Mn), arsenic (As), and nickel (Ni), significantly increases the ecological risk and concerns to aquatic life and human health. This study emphasises the importance of PTE toxicity risk and continuous monitoring for the sustainability of river ecosystems.

## 1. Introduction

The river is the main source of nutrients for life. In recent decades, the proximity of river basins to urban and rural areas has exacerbated the issue of potential toxic element (PTE) contamination in river sediments, water, and aquatic species [1,2,3]. Urbanization, industrialization, and anthropological activities have led to the accumulation of PTEs, posing a momentous threat to aquatic life and river ecosystem health. The Bangladesh Economic Review [4] states that industrial activities have increased in different urban areas. In urban settings, many rivers close to urban centres face heightened risks of contamination from sediment pollution, water pollution, household waste, industrial waste, and chemical discharges [5,6]. Riverbank ecosystems are vital components of aquatic habitats, supporting diverse forms of life, including sediment, soil, flora, and fauna. These ecosystems perform a crucial role in maintaining environmental quality and ecological balance. Particularly, they have experienced a rapid increase in PTE pollution within their river systems due to unregulated industrial growth, inadequate waste management practices, and the discharge of hazardous chemicals [7,8,9]. The sources of PTE contamination are diverse, encompassing industrial waste disposal, mining, smelting, and the improper handling of wastewater and chemical waste [10]. The uncontrolled discharge of vehicle emissions and sewage sludges into rivers is a major contributing factor to the contamination of the aquatic environment [11]. Large volumes of PTEs containing effluent from industries are typically dumped into adjacent water bodies, endangering both the sustainability of the environment and public health [12,13,14]. Based on Theofanis et al. [15], the consequences of PTE contamination extend beyond the immediate aquatic environment. Sediment enriched with PTE pollution becomes part of the aquatic food chain, leading to the accumulation of these toxic elements in living organisms through bio-magnification. The amount of PTEs present in the sediment serves as an important indicator of this equilibrium since it shows how different human activities have affected the health of river ecology. The sediment ecology is threatened by contamination by PTEs [16]. Because of their bioaccumulative nature, toxicity, and environmental persistence, they are regarded as environmental contaminants [17,18]. Above-threshold concentrations of PTEs may accumulate in the biota of riverine ecosystems and have detrimental impacts on both animals and people [19,20]. The potential outcomes of this contamination range from negative impacts on organisms to potential declines in species diversity, abundance, and toxicity risks to human health. Despite the increasing recognition of PTEs, pollution in river sediments worldwide remains a significant gap in ecological research, particularly in the context of developed countries. According to Bashar & Fung [21], the leather and textile industries are potential sources of Pb, Cd, and Ni, which are the principal causes of river sediment pollution. Sediment ecology is negatively affected by Cd and Pb due to anthropological activity. The lack of comprehensive studies addressing PTE contamination across different components of river ecosystems is a critical concern for both environmental and human health due to excessive contamination, and the bio-magnification and toxicity of these pollutants. PTE contamination in sediments may pose a serious ecological concern to urban river ecosystems [22,23,24], although few studies have examined the pollution level of PTEs in river sediment, water, and aquatic species, raising concern over the PTE toxicity risk in ecosystems and for human health. There is very little research on the PTE contamination risk in riverine environments. Therefore, as an initial effort to bridge the research gap, the ecological risks associated with PTE pollution in river sediments, water, and aquatic organisms are comprehensively assessed. River sediment, water, and aquatic species risk may be understood by taking into account the origins, distribution, graphical representation of PTE concentration, and impact of PTE pollution.

### Source of Pollution

River sites in both urban and rural environments are contaminated by a variety of factors, such as construction sites, decomposition of organic matter, logging operations, soil erosion, domestic and industrial chemicals and waste, farming practices, agricultural drainage, and storm-water runoff. River ecosystems face difficult problems as a result of the interaction of various elements. Approximately 15,666 small and 3639 big manufacturing enterprises have been established in Bangladesh, according to the BBS [25]. Proshad et al. [26] reported that a wide range of industries have emerged as a result of rapid industrialization. A glaring concern arises from the discover industries discharging untreated waste directly into the river, raising significant health concerns for the environment and public. Regrettably, insufficient monitoring and inadequate responses have catalysed an escalation in environmental degradation. Yet, the ominous cloud of pollution looms over several river ecosystems, stemming from various sources, including floods, soil erosion, and the discharge of PTEs, chemicals, herbicides, and insecticides, as well as solid and liquid industrial wastes. In the district of Gazipur, Dhaka a staggering 2220 factories operate, with 1222 dedicated to ready-made clothing production. Surprisingly, only 1% of these factories have functioning treatment plants, exposing a vast majority of them to the risk of unregulated waste discharge [27]. In comparison to other factories, the Department of Environment (2016) reported that enforcement activities were higher in fabric-washing factories (38%) and fabric-dying factories (30%). The perilous scale of waste disposal becomes even more apparent when considering the report by Kamruzzaman and Sakib [28], which highlights that around 350 metric tons of harmful garbage are dumped into rivers. Oil refineries add to the burden, with solid waste amounting to 4 tonnes and liquid waste reaching 0.61 million cubic meters annually. The textile dyeing and tanning industries alone contribute significantly, annually generating 113.72 tonnes of solid waste and a staggering 26,250 tons of liquid waste, both of which find their way into river systems, compromising water quality [29]. Soil erosion and mountain cutting stand as significant contributors, accounting for approximately 3% of overall environmental degradation [29]. The DoE [30] attributes approximately 10% of pollution to a combination of factors, encompassing activities such as logging, forest fires, rainwater runoff, and the decomposition of plants and animals. Water pollution is largely caused by these complex biological interdependencies, riverside farming, and agricultural runoff from agricultural regions. Nearshore aquatic habitats provide the most vital ecological functions, from food provisioning to support for human activities, as highlighted by Arikibe and Prasad [31]. This review reveals a concerning interplay between pollution activities and PTE concentrations across distinct river regions. By shedding light on these intricate dynamics, we emphasise the need for targeted interventions and enhanced monitoring strategies to mitigate the escalating ecological risks and human health risks.

## 2. Screening and Systematic Approach

**Figure 1:**
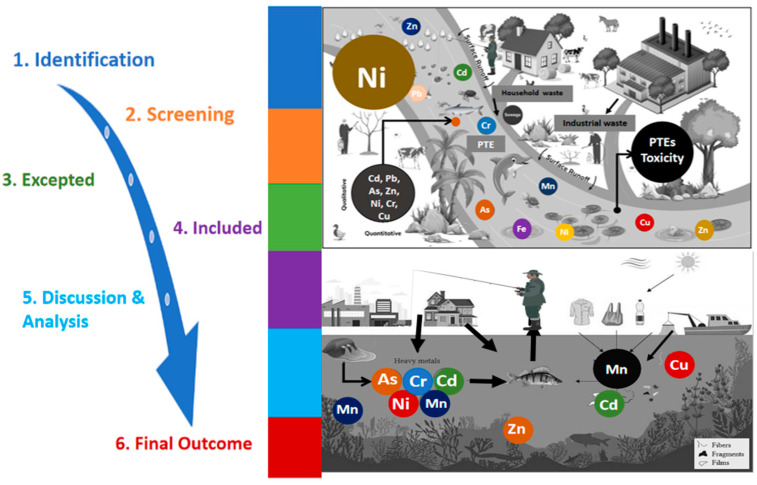
Flow chart of the overall methodology indicating major steps.

The concentration of PTE in sediment, water, and aquatic organisms, as well as concerns about excessive PTE contamination, are the study’s objectives. To achieve our goals, we have chosen and employed a systematic approach, division-wise, utilising information from the Web of Science and Scopus databases, employing relevant search terms such as “river sediment”, “river water”, “aquatic species”, “heavy metal”, “PTEs”, and “element”. For geographical limitation, the selection process involved critically evaluating and choosing the most suitable original research papers in English from the related journal groups such as Elsevier, Springer, Taylor & Francis, PLOS ONE, and others from 2010 to 2024. All the collected information underwent rigorous screening to establish a well-structured portfolio, laying the foundation for the final outcomes of this study and providing directions for future investigations. An outline of the general approach that shows the key procedures and analyses performed in the current investigation is shown in Figure 1. The sediment data visualisation method follows the USEPA standard risk model [32]. We consider the major points for data assembly and analysis. Sediment, water, and aquatic species sample data analysis followed standard protocols [33,34,35].

In Table 1, several PTE indices, indexing factors, and recommendations are described along with their respective contamination standards. Assessment of the pollution risks from PTEs in river sediment, water, and aquatic species are compared based on following the scientific method for monitoring and safety in river ecology. This treasure trove of information subsequently underwent meticulous scrutiny against established Sediment Quality Guidelines (SQGs), affording a comprehensive evaluation of the ecological risks posed by potential toxic element (PTE) accumulation in these vital aquatic systems.

**Table 1:** PTE indices, indexing factors, description, and objectives.

Principal Indexing Factor	Description and Objectives
*C^i^_f_* = *C^i^*/*C^i^_n_* *C_d_* = ∑*C^i^_f_* (*n, i = 1*) PLI = (Cf1 × Cf2 × Cf3 × Cf n)^1/n^ *I_geo_* = Log_2_ ($\frac{Cn}{1:5\times Bn}$) *E^i^_r_ = T^i^_r_* × *C^i^_f_* PER = ∑ *E^i^_r_* ( i = 1) $THQ=\frac{CM}{MRL}$ HI=∑THQ(x) *TCR*$=\frac{EF\times ED\times FIR\times Cf\times CM\times CPSo}{WAB\times TAc}\times 10^{-3}$	PTEs is potential toxic elements. In the equation, *C^i^_f_* is a contamination factor; *C^i^* is the quantified value of PTE in sediments; and the elemental reference value for the same metal follows Taylor [36]; and *C^i^_n_* is the background concentration following Hilton et al. [37] and Karadede and Unlu [38]. *C_d_* is the degree of contamination; PLI is the Pollution Load Index, following Suresh et al. [39]. *I_geo_* is the geoaccumulation index following Müller’s [40] technique. C_n_ is the metal concentration in sediment (n); B_n_ is the metal (n)’s geochemical background value; and the factor 1.5 is the possible variation in background data to lithogenic impacts, following Rabee et al. [41]. *E^i^_r_* is the potential ecological risk index for an individual element. *T^i^_r_* is the biological toxic factor for individual elements and is 5, 30, 2, 5, 1, and 6 for Pb, Cd, Cr, Cu, Zn, and Ni, respectively, following Hakanson [42]. PER is the potential ecological risk index, follows Guo et al. [43] and Hossain et al.’s [22] technique. Non-carcinogenic and carcinogenic: THQ, Target hazard quotient; HI, Hazard Index; TCR, Target Cancer Risk.
Contamination status indication for sediment	Recommendation level: Reference value (mg/kg) Contamination as *I_geo_*: *I_geo_* ≤ 0, no contamination; 0 ≤ *I_geo_* ≤ 1, no contamination to moderately contamination; 1 ≤ *I_geo_* ≤ 2, moderately contaminated; 2 ≤ *I_geo_* ≤ 3, moderately to heavily contaminated; 3 ≤ *I_geo_* ≤ 4, heavily contaminated; 4 ≤ *I_geo_* ≤ 5, heavily to extremely contaminated; 5 < *I_geo_*, extremely contaminated. Contamination as CF: *CF* < 1, low; 1 < *CF* < 3, moderate; 3 < *CF* < 6, considerable; *CF* < 6, very high. Contamination as PLI: *PLI* = 0, indicates excellence; *PLI* = 1, this level is contaminated; *PLI* > 1, the quality is gradually declines.
Contamination Status for water and aquatic species	Recommendation level (mg/kg) Reference value (mg/kg) Toxic level (mg/kg)

## 3. PTE Concentration and Distribution

The concentration and distribution of PTEs in river sediments, water, and aquatic species show regional variations formed by the particular interaction of geographical variables and anthropological activity and influenced by the specific river site and the amount of PTE pollution. A notable reference enriches our understanding, providing the lowest effect levels (LELs) for diverse PTEs in sediment. For instance, Pb exhibits an LEL at 600 mg/kg, Zn at 31 mg/kg, Cu at 120 mg/kg, Cd at 16 mg/kg, Cr at 6 mg/kg, and Ni at 26 mg/kg, among others.

### 3.1. PTEs in Sediments

#### 3.1.1. Lead (Pb)

**Figure 2:**
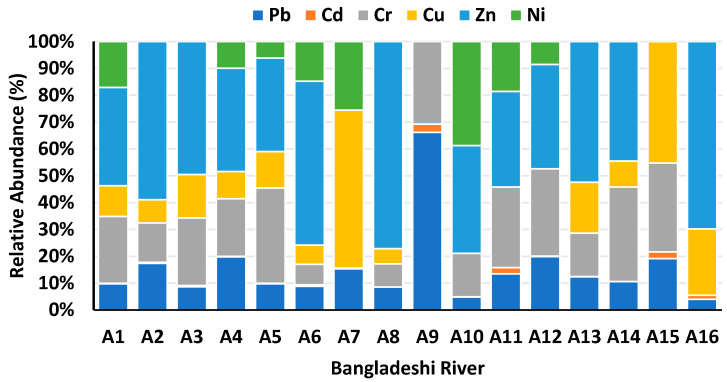
Relative abundance (%) of PTEs in sediment of Bangladeshi river. (Here, A5: Buriganga River; A1: Korotoa River; A9: Karnaphuli River; A2: Meghna River; A3: Shitalakshya River; A4: Rupsha River; A6: Brahmaputra River; A7: Louhajang River; A8: Halda River; A10: Meghna River; A11: Shitalakshya River; A12: Bangshi River; A13: Turag River; A14: Padma River; A15: Dhaleshwari River; A16: Khiru River).

**Figure 3:**
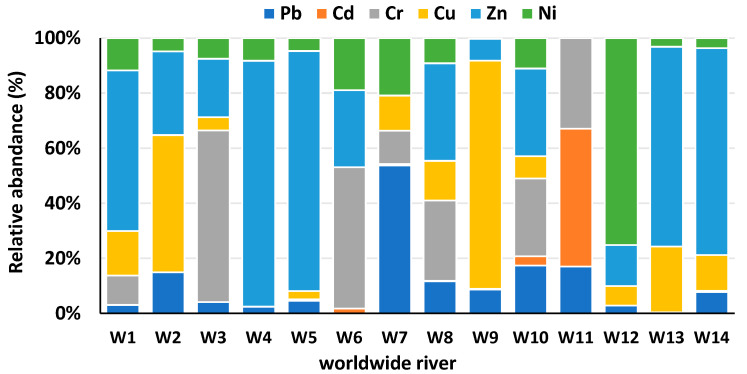
Relative abundance (%) of PTEs in sediment of rivers worldwide. (Here, W7: Symsarna River, Poland; W14: Elbe River, Germany, W10: Ganga River, India; W9: Lubumbashi River, Congo; W11: Okumeshi River, Nigeria; W8: Yellow River, China; W6: Pra River, Ghana; W3: Atoyac River, Mexico; W1: SomesuMic River, Romania; W2: Barma River, Malaysia; W4: Saigon River, Vietnam; W5: Lee River, England; W12: Buyukmelen River, Turkey; W13: Liffey River, Ireland).

Pb is highly toxic to aquatic organisms within contaminated sediments in the ecosystem. High Pb content was found in the Korotoa River (64.67 mg/kg), Rupsha River (62.40 mg/kg), and Buriganga River (477.87 mg/kg), and all values were over background limits (Table 2). Among the rivers in Bangladesh, the Buriganga River sediment exhibits the highest concentration, followed by the Korotoa, Rupsha, Bangshi, Karnaphuli, Turag, Shitalakhya, Dhaleshwari, Meghna, Brahmaputra, and Louhajang rivers. Most river sites surpass the background levels of FAO and SEPAC for Pb, except the Brahmaputra River and Louhajang River sites. Worldwide river sites: other researchers have found Pb pollution in sediment such as in the Yellow River [44] and Xiangjiang River, China [45,46], Gomti River, India [47], Gorges River, Australia [48], Louro River, Spain [49], Symsarna River, Poland [50], and Elbe River, Germany [51] (Table 3). Recently, Proshad et al. [52] reported a high concentration (64.67 mg/kg) of Pb in sediment at the Korotoa River. The Pb contamination in the Buriganga River area stems from the improper disposal of residential and commercial waste, as well as sewage sludge, which has consequently contaminated the sedimentary environment. The highest relative abundance of Pb was found in the Karnaphuli River (Figure 2), with a similar abundance was found in the Symsarna River, Poland (Figure 3). Pb pollution occurred in the river Karnaphuli due to industrial discharge. Specifically, regions such as Suthrapur, Lalbag, and Shyampur within the Buriganga River system demonstrate significant Pb contamination. The FAO recommend Pb concentration of 5 mg/kg in sediment, respectively. The USEPA reported toxicity range from 21 mg/kg to 20 mg/kg for Pb, with the minimum impact of Pb content (31 mg/kg) [53], which raises serious concerns regarding sediment pollution.

Industrial discharges, urban runoff, and atmospheric deposition are common sources of Pb in sediment. Pb toxicity in sediment is a significant environmental concern as it can have adverse effects on aquatic ecosystems and pose risks to human health.

#### 3.1.2. Cadmium (Cd)

Cd is a toxic element, and high levels of Cd were reported in the areas of the Buriganga (5.86 mg/kg), Shitalakshya (5.01 mg/kg), Dhaleshwari (2.08 mg/kg), Khiru (2.05 mg/kg), and Karnaphuli (2.01 mg/kg) rivers in Bangladesh, exceeding the background values of SEPAC (Table 2). The Buriganga River site showed the highest Cd contamination. The Shitalakshya, Dhaleshwari, Khiru, and Karnaphuli River sites’s tapestry tells a fascinating story for Bangladesh. For Cd in sediment, scientifically the lowest impact threshold is 0.6 mg/kg (USEPA).The Hazaribag and Lalbag areas in the Buriganga River basin are conspicuous instances of high levels of Cd contamination, necessitating prompt action to alleviate any risks. Similarly, worldwide, high levels of Cd pollution have been discovered in sediment at the Ganga River, India [54]; Nile River, Egypt [55]; Gardon of Ales River, France [56]; and Lubumbashi River, Congo [57] (Table 3). The USEPA [58] reported Cd toxicity value is 1 mg/kg and guideline limit is 0.61 mg/kg in sediment. A high relative abundance of Cd was reported in the Karnaphuli River in Bangladesh [59] (Figure 2), whereas Raphael et al. [60] identified a comparable abundance at the Okumeshi River in Nigeria (Figure 3). Comparable levels of Cd pollution have been documented around the world, indicating the worldwide reach of the problem and emphasizing the need for efficient mitigation techniques.

#### 3.1.3. Chromium (Cr)

The ecological effects of Cr contamination in sediment ecosystems depend on the oxidation state and concentration of the metal. The most significant amount of Cr in the winter (summer) season is recorded at 158.37 mg/kg (140.53 mg/kg), which is the second highest level of metals for the Buriganga River. The Turag River has the third highest metal levels during the winter (summer) season, with a value of 120.15–30.27 (118.2–28.15) mg/kg. The highest Cr concentration was found in the Buriganga River site, followed by the Korotoa, Bangshi, Rupsha, Turag, Padma, Shitalakhya, Dhaleshwari, Karnaphuli, and Meghna rivers (Table 2). Recently, Proshad et al. [52] found the highest amount of Cr (165.84 mg/kg) in sediment from the river Korotoa. Mohiuddin et al. [61] reported high mean concentrations of Cr (173.4 mg/kg and 709.41 mg/kg) in Buriganga River sites, exceeding the guideline value.The Bangshi River sediment sample is noteworthy due to its Cr content of 33%. Important Cr pollution is found in areas of the Buriganga River system, including the Hazaribag and Lalbag sites. Outside the nation, numerous researchers have discovered Cr pollution in sediment of various rivers, including the Yellow River, China [62], the Pra River, Ghana [63], and the Atoyac River, Mexico (Table 3), which is a global concern for Cr pollution. A high relative abundance of Cr in sediment was found at the Buriganga River in Bangladesh (Figure 2), whereas Rodrigue-Zespinosa et al. [64] discovered a comparable abundance at the Atoyac River in Mexico (Figure 3). The USEPA established a toxicity reference value of 8.10 mg/kg, which raises concerns about Cr pollution in river sediment. Instead, SEPAC [65] reported a background value of 66.80 mg/kg for Cr. Turekian and Wedepohl [66] state the average shale value of Cr is 90 mg/kg whereas the WHO and the USEPA have recommended standards for sedimentary Cr values of 25 and 26 mg/kg, respectively. High levels of Cr pollution indicate a risk for ecology and are of rising concern globally.

**Table 2:** The mean concentration of potential toxic elements (PTEs) (mg/kg) in sediment, pollution sources, and analytical processes are discussed from Bangladesh.

Time	River Name	River study Code	City Code	Pollution Source	Analytical Method	Pb	Cd	Cr	Cu	Zn	Ni
2022	Korotoa River	A1	Bo	AI	ICP-MS	64.67	1.49	165.84	76	243.68	114.13
2021	Meghna River	A2	No	AFA	AAS	12.48	0.28	10.59	6.22	42.41	
2020	Shitalakshya River	A3	Na	IA	FAAS and GFAAS	13.16	0.64	38.39	24.6	75.48	
2020	Rupsha River	A4	K	AI	AAS	62.4	0.56	67.72	31.95	121.35	31.34
2019	Buriganga River	A5	D	IC	ICP-MS	11.405	0.23	41.45	15.93	40.71	7.14
2019	Brahmaputra River	A6	Na	TCDI	FAAS	7.6	0.48	6.6	6.2	52.7	12.8
2019	Louhajang River	A7	T	AI	ICP-MS	4.597	0.083	9. 21	17.727		7.676
2017	Halda River	A8	C	AI	AAS	8.8	0.04	8.84	5.9	79.58	
2016	Karnaphuli River	A9	C	IA	AAS, GF-AAS	43.69	2.01	20.3			
2015	Buriganga River	R11	D	IC	AAS	31.4	1.5	173.4	344.2	481.8	153.3
2015	Meghna River	A10	Nar	IA	AAS	9.47	0.23	31.74		79.02	76.12
2015	Korotoa River	R12	Bo	AI	ICP-MS	58	1.2	109	76		95
2014	Shitalakshya River	A11	Nar	IA	AAS	28.36	5.01	63.22		75	39.22
2014	Bangshi River	A12	T	AA	AAS	59.99	0.61	98.1		117.15	25.67
2013	Turag River	A13	D	AI	AAS	32.78	0.28	43.02	50.4	139.48	
2013	Padma River	A14	D	AI	AAS	11.7		38.91	10.64	49.16	
2012	Dhaleshwari River	A15	S	AI	FAAS	15.79	2.08	27.39	37.45		
2012	Khiru River	A16	M	AA	AAS	5.6	2.05		34.7	97.77	
2011	Buriganga River	R13	D	IC	ICP-MS	477.85	5.86	709.41	224.55	958.15	137.35
2011	Buriganga River	R14	D	IC	AAS	79.8	0.8	101.2	184.4	502.3	
2010	Buriganga River	R15	D	IC	ICP-MS	69.75	3.25	174.53	30.35		200.45
	Toxicity Ref. Value (USEPA)	R16				21.00	1	8.10	28.00	68.00	
	Background Value (SEPAC)	R17				21.90	0.08	66.80	25.50	69.60	33.80
	Average shale Value	R18					0.30	90.00		95.00	

Note: Bo: Bogra, D: Dhaka, No: Noakhali, Na: Narsingdi, Nar: Narayanganj, K: Khulna, T: Tangail, C: Chittagong, S: Savar, M: Mymensingh; ICP-MS: inductively coupled plasma mass spectrometry, AAS: atomic absorption spectroscopy, FAAS: flame atomic absorption spectroscopy, GFAAS: graphite furnace atomic absorption spectroscopy; IC: industrial and commercial; AA: agricultural area; AI: agricultural and industrial; IA: industrial area; TCDI: textile craft and dyeing industries; AFA: agricultural and fishing area. Here, A5 [33], A1 [52], R16 [58], A9 [59], R11 [61], R17 [65], R18 [66], A2 [67], A3 [68], A4 [69], A6 [70], A7 [71], A8 [72], A10 [73], R12 [74], A11 [75], A12 [76], A13 [77], A14 [78], A15 [79], A16 [80], R13 [81], R14 [82], R15 [83].

#### 3.1.4. Copper (Cu)

Globally, diverse Cu concentrations have been recorded, emphasizing the importance of comprehensive monitoring and effective management strategies.Siddique et al. [67] reported Cu concentration in sediment. The maximum concentration of Cu (76 mg/kg) was found in sediment of the river Korotoa. Due to leakage, runoff from nearby unhealthy farms, and the discharge of industrial and municipal trash into the waterway, the Turag River had a higher level of Cu than the Buriganga River. In order of high Cu concentration, the Buriganga River is followed by the Korotoa, Turag, Dhaleshwari, Khiru, Rupsha, Shitalakhya [68], and Louhajang rivers that reveal the background value in Bangladesh (Table 2). Based on USEPA, toxicity value is 28 mg/kg whereas Rupsha river cross the limit [69]. These levels can cause stress and have an impact on aquatic life. Among the rivers, the highest mean concentration (344.20 mg/kg) of Cu in the Buriganga River site exceeded the FAO limit and the background levels of SEPAC. Cu pollution in sediment has been reported worldwide, such as in the Yellow River in China, SomesuMic River, Romani, Barma River, Malaysia, and Liffey River, Ireland(Table 3). A relatively high abundance of Cu was reported at the Louhajang River in Bangladesh (Figure 2), whereas a comparable abundance was found at the Lubumbashi river, Congo (Figure 3). The lowest effect threshold level is 16 mg/kg and in this case Brahmaputra River showed safety zone [70]. Interestingly, notable Cu levels were shown at the Louhajang [71] and Dhaleshwari river sites.

**Table 3:** Potential toxic elements (PTEs) in sediment from different rivers. worldwide.

Sediment (mg/kg)								
River Name	Country	Study Code	Pb	Cd	Cr	Cu	Zn	Ni
Symsarna River	Poland	W7	87.32	0.69	19.76	20.63	0.13	33.88
Elbe River	Germany	W14	122	7.3		206	1190	58
Ganga River	India	W10	151.85	30.01	247.05	70.7	278.61	97.1
Lubumbashi River	Congo	W9	1549	42.9		14,822	1415	55.4
Okumeshi River	Nigeria	W11	0.45	1.32	0.87			
Yellow River	China	W8	24.6	0.3	61.3	30.3	74.6	19.3
Pra River	Ghana	W6		7.27	216.7		118.32	79.9
Atoyac River	Mexico	W3	12		182	14	62	22
SomesuMic River	Romania	W1	12.27	0.35	43.15	65.56	236.82	47.69
Barma River	Malaysia	W2	123			410	250	40
Saigon River	Vietnam	W4	2	0.07			75	6.93
Lee River	England	W5	50	5.64		32.6	946	51.1
Buyukmelen River	Turkey	W12	12.1	0.12		30.6	63.7	323
Liffey River	Ireland	W13		3.25		220	666	29

Note: Here, river study code is W7 [50], W14 [51], W10 [54], W9 [57], W11 [60], W8 [62], W6 [63], W3 [65], W1 [84], W2 [85], W4 [86], W5 [87], W12 [88], W13 [89].

#### 3.1.5. Zinc (Zn)

The amount of Zn in the sediment indicates the level of pollution in the river ecosystem. While Zn is a crucial trace element for many species, high Zn concentrations can impact sediment ecosystems.The highest mean concentration (958.15 mg/kg and 502.3 mg/kg) in the Buriganga River site surpassed the background levels of SEPAC (Table 2). The Zn toxicity value is 68.00 mg/kg for sediment which is exceeded in the Halda River [72], Meghna River [73]. Comparisons with global Zn concentrations highlight the need for a comprehensive approach to mitigate Zn pollution. Islam et al. [74] reported PTE pollution in the urban river area. The Buriganga River continued a pattern of high Zn content, followed by the Korotoa, Turag, Rupsha, Bangshi [76], Khiru, Shitalakhya [75], Halda, and Meghna rivers in Bangladesh. A high Zn concentration (139.48 mg/kg) was discovered at the Turag River site [77] and Padma river site showed safe zone [78]. Ahmed et al. [79] reported PTE pollution in the Dhaleshwari river site. In sediment, the Khiru [80] and Buriganga [81,82] rivers showed high relative abundance (%) of Zn (Figure 2), whereas identified comparable relative abundance in the Saigon River, Vietnam (Figure 3). More patterns were identified in the Hazaribag and Lalbag regions of the Buriganga River system. The USEPA reported average shale value is 95 mg/kg, which raises concerns regarding potential Zn contamination. An increasing number of people worldwide are becoming concerned about Zn contamination that has been found in sediment at the Elbe River, Germany, the Gardon of the Ales River in France, the SomesuMic River, Romania, Lee River, England and Liffey River, Ireland (Table 3).

#### 3.1.6. Nickel (Ni)

Understanding and monitoring Ni contamination in sediment and aquatic environments is crucial for evaluating its potential environmental impacts and implementing appropriate remediation measures to safeguard the health of these ecosystems. A maximum mean value (200.45 mg/kg) of Ni was shown in the Buriganga River site [83], while the Louhajang River site exhibited the lowest concentration (7.676 mg/kg) (Table 2). Ganges river [90] site showed the water and sediment pollution. High concentrations of Ni were observed in the Buriganga, Korotoa, Meghna, Shitalakshya, Rupsha, Bangshi, Brahmaputra, and Louhajang river sites in Bangladesh, exceeding the guideline value. The Turag River had the highest level of Ni (95.1 mg/kg), which is higher than in the Buriganga River. Sediment ecology is becoming more of a concern globally due to the discovery of Ni contamination in sediment at the SomesuMic River, Romania (47.69 mg/kg) [84], Barma River, Malaysia (40 mg/kg) [85]. Recently, the highest level of Ni (114.13 mg/kg) found in sediment along the river Korotoa (Table 2). Remarkable Ni concentrations were found in the Buriganga and Shitalakshya River sites, but the Hazaribag and Lalbag areas of the Buriganga River system had higher Ni levels whereas the Saigon River, Vietnam showed the safety level of Ni concentration [86]. Relatively high Ni concentration discovered in the Lee River, England [87], Meghna River in Bangladesh (Figure 2), whereas Pehlivan [88] found a similar richness at the Buyukmelen River in Turkey (Figure 3). The USEPA and WHO suggested guideline values are 16 mg/kg and 20 mg/kg, respectively, for Ni concentrations in sediment. The dangerous limit for Ni contamination is 16 mg/kg, which is the lowest effective amount of Ni [53]. Also, a high level of Ni was reported at the Ganga River, India, Nile River, Egypt (112 mg/kg), Pra River, Ghana, and Liffey River, Ireland [89] (Table 3).Chronic exposure to Ni leads to unhealthy benthic communities, favouring species that are more tolerant to Ni contamination, altering species composition and disrupting ecosystem dynamics. Even changes in microbial communities affect nutrient cycling, sediment processes, and overall ecosystem health.

### 3.2. Pollution Load Index (PLI)

**Figure 4:**
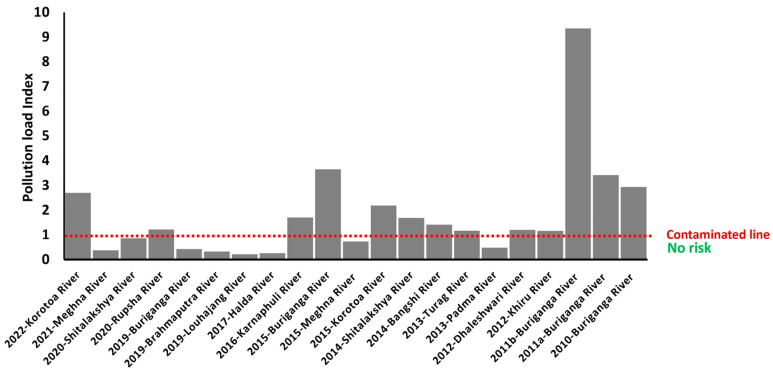
Pollution Load Index (PLI) is represented for different rivers and times.

The sediment pollution status is represented in river areas through an in-depth analysis of PTE concentration. Various PTE results showed novel impacts as an ecological risk when using an integrated potential risk assessment for all metals from river basins. The study utilised the Pollution Load Index (PLI) as an indicator of sediment quality concerning PTEs. The PLI was calculated based on contamination factors for each metal and their corresponding background values. Yi et al. [91] reported PLI in the Yangtze River, China. Similarly PLI value found in the Buriganga and Turag river during the wintertime period ranged from 0.56 to 0.33 and 1.06 to 0.35, respectively, whereas the values for Buriganga and Turag in the summertime period were 0.51 to 0.29 and 1.006 to 0.35, respectively. PLI values exceeding 1 indicated pollution, and values below 1 indicated uncontaminated sediment. The Buriganga, Korotoa, Turag, Rupsha, Shitalakhya, Bangshi, Khiru, and Dhaleshwari rivers in Bangladesh exhibit Pollution Load Indices exceeding the permissible limit of 1, with the PLI range spanning from 0.21 to 9.34 within the research (Figure 4).

The rivers examined displayed PLI values surpassing 1, indicating pollution, with the Buriganga and Shitalakshya rivers exhibiting considerable risk. The PLI value indicated that the Buriganga River sediment was in better condition in 2019 despite being severely contaminated in 2010 and 2011. The significantly raised PLI value of the Korotoa River sediment in 2022 is causing considerable concern for bottom-feeder aquatic species and river ecology. PLI value between 1.64 and 3.89 indicates that the whole stretch of the Ganga River, India, is polluted on many levels. Also observed a PLI range of 0.79 to 1.12 in the Atoyac River, Mexico, indicating PTE contamination.

### 3.3. Geoaccumulation Index (I_geo_)

The geoaccumulation index (*I_geo_*) was employed to assess sediment metal accumulation, comparing concentrations with undisturbed sediment levels. Calculated using specific formulas, the *I_geo_* values indicated pollution levels. Sediment from various river areas exhibited varying levels of contamination, with certain rivers showing moderate Cd contamination and others demonstrating significant Ni contamination.

**Figure 5:**
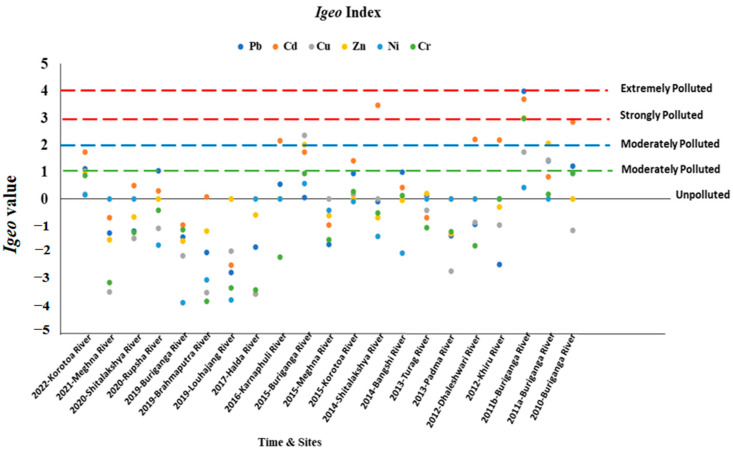
Graph showing geo accumulation index (*I_geo_*) value in sediments at different river sites in Bangladesh.

Examination of the geoaccumulation index (*I_geo_*) values revealed that the Buriganga River site was extremely polluted in 2011 due to Pb, Cd, Cr, and Cu contaminants, whereas in 2019 it showed moderate pollution. The Shithalakshya River site was shown to have unpolluted sediment in 2020 while being heavily contaminated by Cd pollution in 2014. The Korotoa River site was moderately polluted in 2022 due to Cd and Pb pollution while it was in an alarming condition in 2015. In contrast, 2015 recorded moderate pollution attributed to Cu, Zn, and Cd contaminants, while 2019 showed an absence of pollution, highlighting the potential ecological health risks (Figure 5). Other rivers, including the Karnaphuli, Khiru, and Dhaleshwari rivers, exhibit moderate levels of Cd contamination. Sediment sourced from the Korotoa River, the Rupsa River, the Bangshi River, and the Buriganga River sites demonstrates moderate Ni contamination. Furthermore, *I_geo_* values below 0 are observed in significant river sites, indicating an unpolluted status. Further sediment quality analysis is indispensable to manage and ascertain the origins of pollution for potential ecological health. The River Pra, Ghana showed the moderately to extremely polluted with Pb and Ni. *I_geo_* values in the Atoyac River, Mexico indicated unpolluted to moderately polluted with As, Cu, and Pb. The *I_geo_* values in the SomesuMic River, Romania followed as Pb > Cd > Zn > Ni > Cu > Mn > Cr > Fe. Further sediment quality analysis is indispensable to manage and ascertain the origins of pollution for potential ecological health.

### 3.4. Potential Ecological Risk (PER)

**Figure 6:**
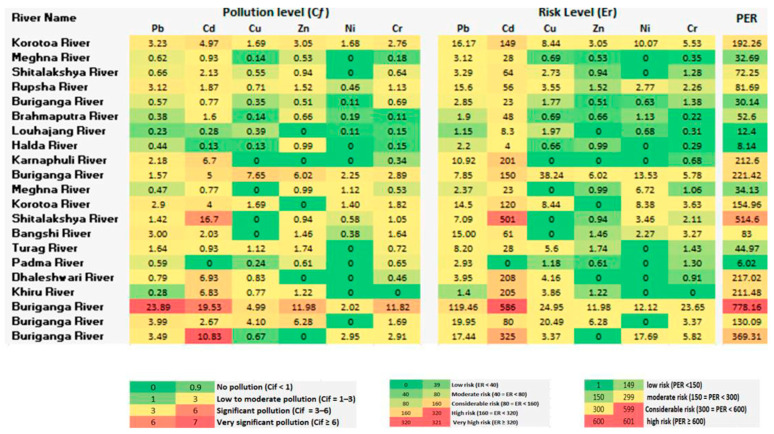
Example pollution levels (*Cf*), risk levels (*Er*), and potential ecological risk (PER) levels in river sediment.

The incorporation of background values enhances the explanation of geochemical data. The contamination factor ($C_{f\;}^{i}$) was employed to assess sediment contamination, and the degree of contamination (*C_d_*) was calculated as the sum of all contamination factors. Their calculation utilized specific formulas, incorporating measured heavy metal concentrations and elemental background values. The degree of contamination was categorized into four levels: no pollution, low-to-moderate pollution, significant pollution, and very significant pollution. Pb contamination exhibited temporal variations, with instances of low-to-moderate pollution and substantial pollution in the Buriganga River site (Figure 6). Similar assessments were made for Cd, Cu, Zn, Ni, and Cr, demonstrating varying pollution levels across different river sites. The Cd and Pb concentration found extremely high levels of contamination, which showed very significant pollution ${(C}_{f\;}^{i}\geq 6)$ in the sediment of the Shitalakshya and Buriganga rivers.

The potential ecological risk index (PER) evaluated the sensitivity of biological communities to toxic substances. Individual elements, area-wise, underwent risk assessment tests based on specific formulas. There was a considerable risk of Pb contamination in the sediment of the Buriganga River site in 2011, whereas a very high risk (ER ≥ 320) was shown for Cd pollution (Figure 6). Except for the Buriganga River, all river sites were revealed as low risk for Pb contamination. A very high risk of Cd contamination existed at the Shitalakshya River site in 2014. The elevated danger of Cd pollution was evident in the rivers Dhaleshwari, Karnaphuli, and Khiru. Additionally, the Bangshi River, Brahmaputra River, Shitalakshya River, and Rupsha River sites were showed considerable risk to moderate risk of Cd pollution. All river sites had a significantly low risk of contamination with Cu, Zn, Ni, and Cr.

We addressed the PER value in the current evaluation of river sediment quality, which is displayed in Figure 6. A moderate-to-extremely high ecological risk was suggested by the PER index level for the sediment from different river sites. Based on the PER value, moderate risk was found in the Korotoa, Karnaphuli, Dhaleshwari, and Khiru rivers, while the Shitalakshya and the Buriganga rivers exhibited significant risk. The PER value showed a moderate risk at the Korotoa River site in 2022. The Buriganga River (PER ≥ 600) and the Shitalakshya River (300 ≤ PER < 600) sites showed higher potential ecological risk than other rivers. Both rivers are located in the industrial area of Dhaka. Pb and Cd, especially, enhanced the potential ecological risk. At these two river locations, different sources contribute to the PTE pollution of the sediment. The PER values in the Atoyac River, Mexico showed decreasing order: As (80) > Pb (70) > Cu (57) > Ni (38) > Cr (26) > Zn (11).

## 4. PTE Pollution Status in River Water

The majority of urban river water is contaminated by debris from anthropogenic activity, chemical discharge from industrial zones, and wastewater. In Bangladesh, the Louhajang River water showed safe level of PTE contamination [92]. Many researchers have significantly evaluated PTE concentrations in different river water [93,94,95,96,97,98,99] (Table 4). During the winter the mean concentration of PTEs in Buriganga river water followed Fe > Cr > Ni > Zn > Cu > Pb, but in the summer, the order was Fe > Cr > Zn > Ni > Cu > Pb. The highest level of Fe in the Buriganga River was recorded to be 20.43 mg/L in wintertime but dropped to 13.49 mg/L in summertime. The readings in the Turag River were 10.16 mg/L and 7.1 mg/L, respectively. Among the rivers on the list, Buriganga, Turag, Dhaleshwari, Korotoa, Karnaphuli, and Bangshi all had Cr contamination in their river water. High concentrations of Ni were discovered in water from the Lee River, England and the Ganga River, India (Table 4).The Turag river water showed high level of PTE contamination [93], whereas Shitalakhya River water showed the safe level of PTE pollution [94].

The water of Dakatia, Khiru, Old Brahmaputra, and Karnaphuli all had signs of Mn poisoning. The levels of Fe contamination in the Karnaphuli, Dhaleshwari, Turag, and Buriganga waterways rose. The Buriganga and Korotoa Rivers exhibited high levels of Ni contamination, above Bangladesh’s water quality standard guideline values. Water from the Bangshi, Korotoa, and Halda rivers had high levels of Cu contamination. The Karnaphuli, Korotoa, and Halda rivers were found to be contaminated by As. Water from the Karnaphuli River exhibited high levels of Pb and Cd contamination. Similarly, high levels of Pb and Cu pollution in water were shown in the Ganga River in India, the Lee River in England, and the Yangtze River in China (Table 4). The Buriganga [102] and Dakatia [103] river water showed higher Mn content than the normal content that reported by BDSW. The water of the Bangshi River had a Cr concentration of 0.093 mg/L [104], whereas BDSW [105] recorded 0.05 mg/L.WHO [106] found that the content of Cr in water is 5 µg/L.

## 5. PTE Pollution Status in Aquatic Species

Numerous researches have discovered PTE contamination in fish, crabs, and molluscs, and small amounts of PTEs may build up in the river environment in fish and other aquatic species. High levels of Cr were found in fish and shellfish of the river Buriganga and Turag (Table 5). Safe level of Pb and Cd content was reported in fish from Kelantan River, Malaysia [107]. Khan et al. [108] reported high levels of Pb, Cd, and Cr in freshwater fish at the Swat River, Pakistan. Scientifically, many researcher reported notable level of PTE concentration in aquatic species [109,110,111,112,113,114,115,116,117,118,119] from the river ecosystem. PTE pollution in the sediment affects the bottom-feeder aquatic species (*A. coila*, *G. youssoufi*, *M. pancalus*, *H. fossilis*, *C. punctata*, *M. vittatus*). Also Concentration of PTE reported in crustaceans (*M. rosenbergii*), and molluscs (*I. exustus*) (Table 5). Human ingestion of these type of species from the polluted river habitats increases the risk of cancer. Health hazards have been connected to PTE contamination of fish and shellfish from sediment areas. Cu exposure from sediment caused physiological stress in organisms, which impacted their food chain, general well-being, and cellular functions. Diseases and other environmental stresses are more likely to affect organisms due to Cu pollution. Fish species from the Dhaleswari, Buriganga, and Turag rivers showed high levels of Cr contamination. Fish species in the Turag River displayed high levels of Fe contamination. All of the mentioned rivers’ fish, crustaceans, and mollusc species had high levels of Zn contamination, except the Turag-Tongi-Balu River. The fish species found in the Karnaphuli and Bangshi rivers had high levels of As pollution.The majority of the fish and mollusc species in the river exhibited significant levels of Pb pollution. Fish and crab species from the Buriganga, Turag-Tongi-Balu, and Bangshi rivers exhibited high levels of Cd contamination.

## 6. Effects on Human Health

It is not possible for the public to completely avoid the potential health concerns associated with consuming aquatic animals that have accumulated PTEs. These PTEs disturb the food pyramid and endanger human health by creating cancer and human organ disorders.Hazard index (HI) value of freshwater fish greater than 1 (HI > 1) indicates non carcinogenic risk to human health (adult). China’s freshwater fish and crabs’ hazard index (HI) value is in the safe region (<1) in Table 6. High values of hazard index (HI >1) reported in *L. rohita* (3.78) (Bangladesh), *O. niloticus* (4.53) (Egypt), *P. notialis* (6.1) (Ghana), *M. armatus* (13.71) (India), and *P. laevis* (1.5) (China) from the river ecosystems that related to PTE pollution.

Many researchers have reported cancer risk (CR) values of Ni, As, Cd, and Pb, as shown in Table 7 for different aquatic species. CR values ranging from 10^−5^–10^−4^ for all metals are related to moderate cancer risk, and 10^−3^–10^−1^ indicates cancer risk in human health. Neurological diseases in humans are caused by high amounts of Cd, Pb, and Cr [120,121]. Eye yellowing is a result of As, Cd, and Pb pollution and decreased thyroid hormone synthesis [122], elevated heart illnesses [123], and exacerbated peripheral vascular disease [124]. Contamination with Cr, As, Cd, and Pb leads to haemolysis in illnesses of the cardiovascular system [125,126]. Cd pollution causes illnesses such as pulmonary system fibrosis [127] and bone degradation [128]. Long-term exposure to Cd has been linked to harmful health outcomes, such as lung and kidney damage, as well as an increased chance of developing several malignancies.

**Figure 7:**
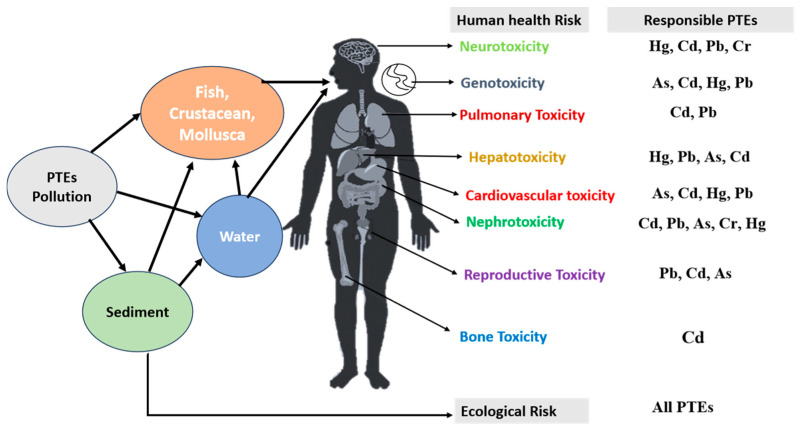
A model of PTE pollution in river sediment, water, and aquatic species, and showing possible toxicity risk in human health for different PTEs.

According to Kim et al. [129], liver disorders and cirrhosis are caused by Pb, As, and Cd pollution. Contamination with Cd, Pb, As, and Cr can cause skin illness and gastrointestinal upset [130]. Tiredness, feeling sick, haemophilia, and electrolyte imbalance have all been linked to Zn toxicity. Long-term exposure to inorganic As can have negative consequences on the neurological system, haematological system, skin, liver, gastrointestinal tract, respiratory tract, and cardiovascular system [131].Women will have difficulties becoming pregnant [132,133] due to Pb, Cd, and As contamination. For human health, the NYSDOH [134] categorises cancer risk (CR) as follows: if CR ≤ 10^−6^ = low; 10^−4^ to 10^−3^ = moderate; 10^−3^ to 10^−1^ = high; ≥10^−1^ = very high. We have found different toxicity effects on human health due to PTE pollution in wetland ecosystems (Figure 7).

**Table 6:** Hazard index (HI) values reported for aquatic species.

Species	Study Code	Hazard Index (HI)	Country
*L. rohita*	H1	3.78	Bangladesh
*C. punctata*	H1	1.72	Bangladesh
*C. batrachus*	H1	1.17	Bangladesh
*H. fossilis*	H1	1.10	Bangladesh
*Cynoglossus* sp.	H5	23.57	Nigeria
Fish sp.	H6	1.69	China
*C. fluminea*	H7	1.67	Bangladesh
*C. amnicola*	H8	8.34	Nigeria
Clam	H9	1.148	China
Freshwater fish	H10	0.558	China
Freshwater crab	H10	0.092	China

Note: here, H1 [35], H5 [135], H6 [136], H7 [137], H8 [138], H9 [139], H10 [140].

**Table 7:** CR value of PTEs from consumption of aquatic species.

Species	Study Code	Potential Toxic Elements (PTEs)				Country
		As	Pb	Ni	Cd	
*L. rohita*	A1	2.86 × 10^−5^	1.76 × 10^−6^	3.00 × 10^−4^	8.32 × 10^−5^	Bangladesh
*C. punctata*	A1	3.08 × 10^−5^	5.69 × 10^−6^	9.73 × 10^−5^	3.00 × 10^−3^	Bangladesh
*H. fossilis*	A1	3.52 × 10^−5^	5.20 × 10^−6^	3.00 × 10^−4^	6.00 × 10^−4^	Bangladesh
*Cynoglossus* sp.	B1		4.02 × 10^−8^	2.35 × 10^−5^	6.48 × 10^−6^	Nigeria
*C. fluminea*	C1	1.00 × 10^−4^	1.00 × 10^−6^	2.00 × 10^−4^	2.00 × 10^−3^	Bangladesh
*C. amnicola*	F1	1.09 × 10^−7^	1.66 × 10^−8^		4.64 × 10^−8^	Nigeria
*L. camtschaticum*	G1	1.60 × 10^−4^				China
*C. farreri*	H1		7.44 × 10^−8^		1.93 × 10^−3^	China
*C. ariakensis*	H1		5.87 × 10^−8^		2.38 × 10^−4^	China
*S. constricta*	H1		7.26 × 10^−8^		3.94 × 10^−5^	China
*C. gariepinus*	K1	2.00 × 10^−3^	9.90 × 10^−6^			Egypt
*O. niloticus*	K1	1.40 × 10^−3^	1.20 × 10^−5^			Egypt
*O. aureus*	K1	1.50 × 10^−3^	9.80 × 10^−6^			Egypt
*T. zillii*	K1	1.70 × 10^−3^	9.60 × 10^−6^			Egypt
*H. molitrix*	P1		7.11 × 10^−6^	2.55 × 10^−5^		China
*C. idellus*	P1		1.97 × 10^−6^	1.85 × 10^−4^		China

Note: here, A1 [35], B1 [135], C1 [137], F1 [138], G1 [141], H1 [142], K1 [143], P1 [144].

## 7. Research Perspective: Implications and Future Directions

The assessment of pollution risk through river sediments, water, and aquatic species is highly important for ecosystems. This review found different river sites have a spectrum of pollution levels, ranging from moderate to strong ecological risk. Notably, the Buriganga River and Shitalakshya River sites stand out as areas of heightened ecological risk, surpassing other locations. This disparity underscores the urgency of in-depth investigations and continuous monitoring in these critical zones, necessitating further research to comprehend their intricate dynamics and the potential ramifications for river ecology. Furthermore, the examination of geoaccumulation index values across diverse river sites has consistently revealed levels exceeding background sediment values. Identified potential ecological risks (PERs) at the Buriganga River and Shitalakshya River sites flag them as high-risk zones, raising concerns about their impact on benthic organisms, aquatic species (fish, crustaceans, molluscs), and even human health. These findings underscore the interconnections of ecological well-being and human health safety, highlighting the imperative of comprehensive ecosystem management. The observed spatial disparities in PTE pollution and sediment accumulation underline the intricate environmental dynamics within river ecosystems. The breach of LELs for specific metals in certain regions signals a potential menace to the ecological equilibrium of these areas. Cd and Pb concentrations exceeding typical shale values raise alarm bells, demanding immediate attention to mitigate potential adverse repercussions on the environment and human health.

This review offers crucial insights into the ecological risks posed by PTE accumulation in river sediments, water, and aquatic species. The divergent pollution levels across various river sites and the intensified health risk in specific regions underscore the necessity for ongoing research on sediment, water, and aquatic species; proactive surveillance; and strategic interventions to ensure river ecosystems’ long-term vitality and sustainability. The findings highlight the pivotal role of interdisciplinary collaboration and well-informed decision making in mitigating the potential impact of PTE contamination on the environment and human populations.

Potential toxic elements (PTEs) are released into the riverbank ecosystem and pollute river sediment, water, and aquatic species as a consequence of several industrial processes, including municipal trash, fuel refining, smelting, tannery waste, and chemical waste. An ecological risk assessment of sediment encompasses evaluation of potential adverse effects stemming from contaminants or stressors on sediment ecosystems and their inhabitants. Sediments serve as repositories for diverse pollutants, including PTEs, organic chemicals, and nutrients. Accumulation of these contaminants over time can endanger aquatic life, benthic organisms, and even humans through the food chain. This review details the extent of sediment, water, and aquatic species pollution in the river area. The presence of sediment pollution amplifies ecological risks, with approximately 83% of water bodies exhibiting high pollution rates. Across all the studied rivers, the average concentration of various PTEs (Pb, Cd, Cr, Cu, Zn, Ni) in sediment exceeded recommended Sediment Quality Guidelines (SQGs) ranges, following the order: Zn > Cr > Ni > Cu > Pb > Cd. Pollution Load Index (PLI > 1), PER index, and geoaccumulation (*I_geo_*) index values collectively designate the Buriganga, Turag, Korotoa, Karnaphuli, Rupsha, and Shitalakshya river sites as heavily polluted due to PTE contamination. Effective management of pollutants is of paramount importance for minimizing the ecological impact of hazardous industrial materials and contaminants. Consequently, this study identifies, discusses, and underscores potential ecological risks using various risk assessment methodologies and established risk thresholds. There are concerns about human health due to PTEs’ contamination of water and aquatic species. Monitoring the river region’s environment necessitates continued research on sediment, water quality, and pollution dynamics, serving as a valuable foundation for future studies in this domain.

## 8. Conclusions

Anthropological activities and industrial discharges are causes of the alarming levels of PTE pollution in urban river ecosystems. Highly industrialised sites and residential settlements next to release sources raise exposure concerns. Our findings showed that risk models significantly underestimate the quantities of PTEs in river sediment. Concerning levels of PTEs were found in the sediment, water, and aquatic species, which has a significant impact on the environment and human health. Human health toxicity risks were emphasised by PTEs, especially As, Pb, and Cd contamination. Also indicated carcinogenic risk through CR value. An hazard index value greater than 1 poses risk to human health. High content of Mn poses non-carcinogenic risk in human health. A number of rivers showed ecological risk associated with PTEs. These findings highlight the value of innovation as a tool for regulatory monitoring for precisely identifying hazardous exposures, pollution levels, and ecological risk levels in river sediments. The PLI, *I_geo_*, and PER revealed that river sediments exhibit significant Cd and Pb contamination, particularly in the Buriganga River, posing serious consequences and risks to human health and aquatic life. The PER for the Shitalakshya and Buriganga rivers is very high, indicating severe ecological risks in addition to medium-level contamination of the Dhaleshwari and Khiru rivers. The environmental risk was elevated by PTE pollution in the riverbed region, which demonstrated moderate-to-significant ecological risks as well as contamination of water and aquatic species. Specifically, the additive effects exacerbated by the presence of Mn, Zn, Pb, Cd, and Ni in aquatic species; Fe, Ni, and As in water; and Pb, Cd, Cr, and Ni in sediment further aggravate ecological risks in aquatic ecosystems, emphasizing the crucial need for continuous monitoring and stricter regulations to safeguard the sustainability of river ecosystems and protect public health we can make significant progress in mitigating the ecological and human health risks by continuous monitoring, combined with proactive management and policy interventions, water treatment, sustainability and health of these vital water resources. In addition, strengthening the environmental regulations to enforce stricter limits on PTE emissions from industrial and urban sources and the implementation of reformed policies could incentivise the adoption of cleaner technologies and penalise non-compliance. Finally, educating the masses about the ecological consequences of PTE contamination and encouraging industries to adopt sustainable practices, such as closed-loop systems and waste minimization techniques, could significantly reduce the discharge of PTEs into the environment.

## Figures and Tables

**Table 4:** Pollution source and PTE concentrations in river waters from different areas.

Time	Water Body Name	River Water Study Code	Country/City Code	Pollution Source	Cr	Mn	Fe	Ni	Cu	Zn	As	Pb	Cd	Unit
2021	Buriganga River	E1	B/D	TDIMW	1.99	-	12.31	1.05	0.685	1.065	-	0.27	-	mg/L
2021	Turag River	E2	B/D	TDIMW	0.61	-	6.995	0.965	0.75	1.6	-	0.32	-	mg/L
2021	Halda River	E3	B/C	IESG	0.032	0.08	0.48	-	1.751	0.2275	1.205	0.067	0.036	mg/L
2021	Karnaphuli River	E4	B/C	WBWMS	0.006	1.53	4.34	0.018	0.007	0.059	-	0.022	0.017	mg/L
2021	Louhajang River	E9	B/D	IMHA	0.0052	-	-	0.0041	0.0062	-	0.0059	0.0051	0.0004	mg/L
2021	Dhaleshwari River	E10	B/D	IMDW	0.71	-	-	0.62	-	0.18	-	-	0.19	mg/L
2021	Meghna River	E11	B/C	IMDW	0.045	-	-	-		-	0.024	0.009	0.018	mg/L
2021	Sela River	E12	B/K	OSAF	0.0346	0.035	0.0734	0.1481	0.0236	0.028	-	0.0837	0.0307	mg/L
2020	Ganges River	E13	B/K	Geogenic	0.0007	0.0002	0.08	0.005	0.0067	0.089	0.0021	0.0026	0.0001	mg/L
2019	Old Brahmaputra	E6	B/D	IDID	0.01	1.44	-	0.44	0.12	0.01	-	0.11	0.001	mg/L
2019	Ganges River	E8	B/R	IMA	0.038			0.004	0.012	0.03	0.003	0.009		mg/L
2018	Korotoa River	E14	B/R	MIEAR	1.13	-	-	1.33	3.02		2.62	0.81	0.75	mg/L
2018	Balu River	E15	B/D	IMDW	0.0044			0.0132			-	0.0186	0.003	mg/L
2017	Passur River	E16	B/K	GS	0.02		0.27		0.02	0.01				mg/L
2016	Turag River	E17	B/D	IMDW	0.0558	0.7085	2.606	0.1309	0.2308	0.2952	0.0034	0.0146	0.0143	mg/L
2016	Shitalakhya River	E18	B/D	IMDW					0.0242			0.023	0.0073	mg/L
2016	Karnaphuli River	E5	B/C	WBWMS	69.56						23.36	9.85	6.46	µg/L
2015	Buriganga River	E19	B/D	TDIMW	0.114	0.157	0.612	0.15	0.239	0.332	0.134	0.119	0.059	mg/L
2015	Dakatia River	E20	B/C	USF	0.003	0.334	0.218		0.033	0.114		0.006	0.001	mg/L
2013	Bangshi River	E21	B/D	TDAD	0.093	0.088		0.035	1.05	3.32	0.024	0.108	0.007	mg/L
2012	Khiru River	E7	B/M	IMDW		0.167			0.0043	0.007		0.0221	0.128	mg/L
	Ganga River	W10	India/		70.16			61.11	43.72	71.37		80.55	11.41	µg/L
	Lee River	W5	England/					55.1	46.7	62.7		13.3	0.44	µg/L
	Yangtze River	W15	China/		1.3				2.8	31		2	0.4	µg/L
	BDSW	E22			0.05	0.1	0.3	0.1	1	5	0.05	0.05	0.005	mg/L
	WHO	E23			5						10	10	3	µg/L
	TRV				11						150	3	2	µg/L

Note: B-Bangladesh, D: Dhaka, C: Chittagong, K: Khulna, M: Mymensingh, R: Rajshahi. TDIMW: textile and dyeing industries, municipal waste; IDID: industrial, domestic, and irrigation discharges; IMDW: industrial and municipal discharged water; IMHA: industrial, municipal, and household activities; TDAD: textile, dyeing, and apparel industries, DEPZ; USF: urban sewage, different types of factories; IESG: industrial effluents, sewage, geogenic reason; WBWMS: wastewater, bedrock weathering, metal smelting; GS: geological sources; OSAF: oil spillage, accident, factories; MIEAR: municipal, industrial effluents, agricultural runoff; BDSW: Bangladesh’s standard of water quality. Here, E1 [22], E2 [22], E3 [23], E4 [24], W10 [54], E5 [59], E6 [70], E7 [80], E8 [90], W5 [87], W15 [91], E9 [92], E10 [95], E11 [96], E12 [97], E13 [98], E14 [99], E15 [100], E16 [101], E17 [93], E18 [94], E19 [102], E20 [103], E21 [104], E22 [105], E23 [106].

**Table 5:** PTE concentration (mg/kg) in fish, crustaceans, and molluscs from different rivers.

Time	Name	Country (City Code)	Study Code	Aquatic Species	Analytical Method	Cr	Mn	Fe	Ni	Cu	Zn	As	Pb	Cd
2024	Turag-Tongi-Balu	Bangladesh (D)	S1	*H. fossilis*	ICP-MS and FAAS	1.74	26.32	19	1.34	0.2	6.39	0.16	4.17	0.67
2023	Swat River	Pakistan	S2	fish	ICP-MS	14.59			11.80	8.59	116.83		1.95	0.29
2022	Dhaleswari River	Bangladesh(D)	S3	*H. fossilis*	AAS	22.6			5.55		98.19		7	
2021	Buriganga River	Bangladesh (D)	S4	*H. fossilis*	AAS	187.07		39.07	3.01	3.51	35.12		5.07	
2021	Turag River	Bangladesh (D)	S5	*H. fossilis*	AAS	70.18		45.1	12.18	6.03	68.25		6.22	
2019	Karnaphuli River	Bangladesh(C)	S6	*P. chinensis*	ICP-MS	3.59				13.1		5.03	14	0.44
2019	Karnaphuli River	Bangladesh (C)	S7	*T. ilisha*	GF-AAS and AAS	0.65						1.22	0.67	0.15
2016	Buriganga River	Bangladesh(D)	S8	*L. rohita*	ICP-MS	18.84	125.81		6.64	18.77	251.69	0.73	6.98	0.04
2015	Buriganga River	Bangladesh(D)	S9	*M. pancalus*	ICP-MS	7.18	25.65		1.6	11.66	165.1	0.22	3.17	0.01
2015	Buriganga River	Bangladesh(D)	S9	M. rosenbergii *	ICP-MS	1.59	35.25		0.44	575.34	187.04	1.19	0.51	1.51
2015	Buriganga River	Bangladesh(D)	S9	I. exustus **	ICP-MS	16.05	319.66		5.75	16.47	58.56	1.02	4.55	0.05
2014	Kelantan River	Malaysia	S12	fish	GF-AAS								0.072	0.05
2012	Bangshi River	Bangladesh(D)	S13	*H. fossilis*	AAS	0.71	26.11		4.11	14.17	203.19	6.24	7.71	0.31
	USFDA (FSG)		S14			13			80					
	FAO		S15			1	1			10	30	1	2.5	0.2
	WHO		S16							30			2	
	FAO/WHO		S17									1		
	IOM		S18					40–45						
	EC (EU)		S19			1				3			0.1	0.05

Note: D: Dhaka, C: Chittagong, * crustaceans, ** molluscs. Here, S7 [6], S4 [22], S5 [22], S1 [35], S12 [107], S2 [108], S3 [109], S6 [110], S8 [111], S9 [112], S13 [113], S14 [114], S15 [115], S16 [116], S17 [117], S18 [118], S19 [119].

## Data Availability

Data will be made available on request.
